# Anesthetic Management of Thoracoscopic Pulmonary Cystectomy in a Patient With Fontan Circulation and Disturbed Lung Inflation During Leakage Testing: A Case Report

**DOI:** 10.7759/cureus.64391

**Published:** 2024-07-12

**Authors:** Ayaka Higashi, Mao Kinoshita, Kazuki Sudo, Hiroshi Ueno, Teiji Sawa

**Affiliations:** 1 Department of Anesthesiology, Kyoto Prefectural University of Medicine, Kyoto, JPN; 2 Department of Anesthesiology, Kyoto prefectural university of medicine, Kyoto, JPN

**Keywords:** negative pressure management, thoracic catheter, leak testing, one lung ventilation, fontan circulation

## Abstract

Congenital heart disease may require multiple cardiac surgeries during childhood. Subsequent non-cardiac surgeries increase the perioperative bleeding and hypoxia because of changes in circulation. An 18-year-old male patient with a history of multiple cardiac interventions, including Fontan surgery, underwent a thoracoscopic right lung suture and coverage for recurrent right spontaneous pneumothorax under general anesthesia with one lung ventilation (OLV). The superior dorsal and inferior lobes, which were inflatable before surgery, failed to expand during leakage testing. The trachea’s condition was examined using a flexible bronchoscope, and no obstructions were found. A thoracic drainage catheter was inserted, and the lower lobe was dilated from outside the body using negative pressure control in a sealed environment. In the patient with previously treated Fontan circulation, both lungs were expanded by inserting a thoracic catheter during thoracoscopic right lung suture and maintaining negative external pressure.

## Introduction

Recent advances in diagnostic and treatment modalities have increased the survival rates of many patients with congenital heart disease, allowing them to reach adulthood; more than 500,000 of these patients have already achieved this in our country [1]. In this context, the advancement of surgical treatment and the overall improvement of medical management have increased the number of patients who have undergone a Fontan procedure for anamnesis and are undergoing additional noncardiac surgery [2,3].

Perioperative complications in patients with Fontan circulation occur in 31% of other types of surgery, with a postoperative mortality rate of one in every 39 cases [4]. Thus, perioperative anesthetic management requires special attention. In particular, disrupted blood flow in the pulmonary circulation can contribute to the development of heart failure, even when the ventricular preload is increased due to increased pulmonary arterial resistance (PVR). Thus, the maintenance of the systemic ventricular preload is extremely sensitive to changes in capacitance load and pulmonary vascular resistance in this pathologic state [5].

There have been few cases of OLV in patients with Fontan circulation reported in the literature, and most of the data are limited. We present not only the respiratory and circulatory management in thoracic surgery of a patient with Fontan circulation but also the anesthetic of a thoracoscopic pulmonary cystectomy in which lung expansion was difficult to achieve using a lung leak test. The patient provided written informed consent for the publication of this case report and accompanying images.

## Case presentation

An 18-year-old male patient with height of 163 cm and weight of 45.6 kg was scheduled for thoracoscopic pulmonary cystectomy due to right-side pneumothorax. The patient had undergone several cardiac surgeries as a child, including Fontan for hypoplastic right heart, severe tricuspid and pulmonary valve stenoses, patent ductus arteriosus, and foramen ovale. Chest X-ray showed no cardiomegaly, and electrocardiogram revealed no abnormalities. Blood tests revealed a platelet count of 100,000/uL. The renal function was normal, and there were no electrolyte abnormalities. The patient received warfarin, aspirin, and heparin bridging therapies before surgery and had normal coagulation function on the day of the procedure.

The intervention was performed under general anesthesia with wound infiltration analgesia. Intravenous patient-controlled analgesia was the planned postoperative pain relief. General anesthesia was induced using a rapid sequence that included 3.0 ug/mL propofol target-controlled infusion (TCI), 100 ug of fentanyl, and 0.2 ug/kg^−1^/min^−1^ of remifentanil and was maintained with propofol TCI. We selected induction agents based on their ability to affect pulmonary blood flow and cardiac output. The OLV was inserted through a left-sided double-lumen tube (35 Fr. Parker Double-Lumen Endobronchial Tube; Japan Medicalnext Co., Ltd., Osaka, Japan). The latter was performed under general anesthesia, with perioperative management focusing on the hemodynamics of Fontan circulation using intra-arterial pressure measurements (FloTrac; Edwards Lifesciences, CA, USA), central venous catheterization from the right internal jugular vein, and transesophageal echocardiography (TEE). Central venous pressure was kept between 10 and 15 mmHg, and the cardiac contractility and respiratory variability of the inferior vena cava were tracked by TEE as needed for circulatory control. As a result, intraoperative respiratory and circulatory dynamics remained stable.

**Figure 1:**
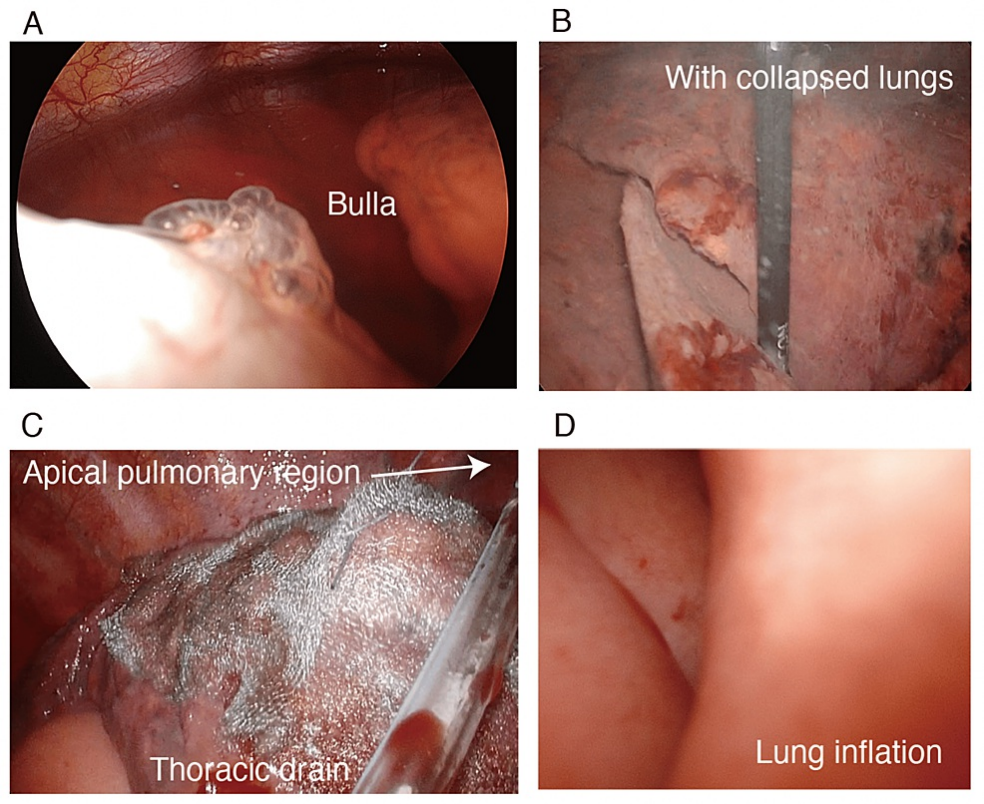
Thoracoscopic findings. The lungs did not expand during a leakage test performed under pressure from a tracheal tube, but lung expansion was achieved by inserting a thoracic drainage catheter.

**Figure 2:**
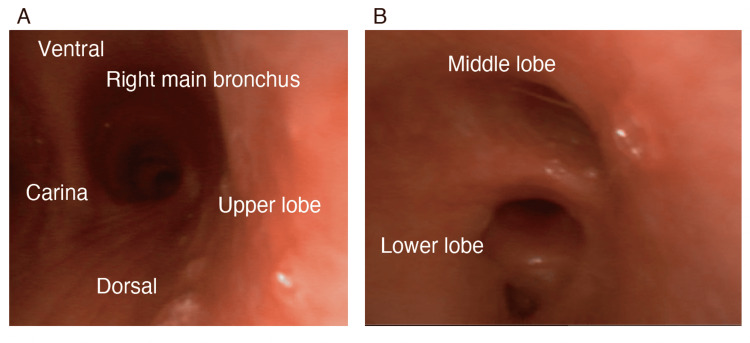
Bronchoscopic findings. Bronchoscopic findings in the trachea during surgery. The right bronchial branch was not obstructed.

Intraoperative findings revealed a 1 cm bulla in the right S6 area, with air leaking from the same location (Figure 1A, 1B). A right lung suture was performed, and absorbable tissue reinforcement was applied to the apex of the lung and the repaired right S6 area to prevent pneumothorax recurrence. In addition, the upper and middle lobes of the anterior mediastinum were dissected due to the presence of adhesions. The airway pressure was then set to 15 mmHg, and both lungs were pressurized to check for air leaks. The right dorsal upper and lower lobes, which were inflatable before the procedure, did not expand under pressure. The trachea’s condition was examined using a flexible bronchoscope and found no obstruction (Figure 2A, 2B). A thoracic drainage catheter was inserted through the operative field to distend both lungs without increasing airway pressure, and negative pressure control of −10 cmH_2_O between the sealed thoracic cavity and external body resulted in lower lobe expansion (Figure 1C, 1D). The patient was extubated following intervention and was transferred to the intensive care unit after his respiratory and circulatory status had stabilized. The operative time was 3 hours and 29 min, and anesthesia took 6 hours and 14 min. The patient lost 5 mL of blood, produced 520 mL urine, and received a 1575 mL crystalloid infusion. The thoracic catheter was removed on the first postoperative day, and the patient was discharged on the third day.

## Discussion

OLV has several negative effects on the health of patients with Fontan circulation. The physiological changes associated with OLV, such as ventilation failure, hypercarbia, hypoxia, and hypoxic pulmonary vasoconstriction (HPV), reduce blood flow within the pulmonary circulation by increasing the PVR [4,6,7]. Furthermore, elevated intrathoracic pressure caused by positive-pressure ventilation during OLV may prevent preventricular venous return in this patient population [7]. Although there are only a few case reports in the current literature, there have been some Fontan circulation failures [4,7]. In this case, we could ensure the patient’s safe management during the perioperative period. Furthermore, during anesthesia and thoracoscopic right lung suture in a Fontan surgery patient, the lung did not expand despite the absence of endotracheal obstruction. We had a case in which both lungs were dilated by inserting a thoracic drainage catheter and monitoring negative pressure from outside the body.

Although there are sparse case reports of OLV in patients with Fontan circulation, there have been instances in which the latter was disrupted [4,7]. In this case, we could provide safe perioperative management. In addition to regulating the circulation, we altered oxygenation and CO_2_. Increased PVR during isolated lung ventilation was expected to cause hemodynamic compromise, but this could be managed using central venous pressure and transesophageal echocardiographic monitoring, as well as circulatory agonists (dobutamine). During the perioperative period, there were no disturbances in cardiac function or severe atrioventricular regurgitation, monitoring of CVP, and arterial pressure measurements allowed for effective guidance of the administered infusion. CVP’s values typically ranged between 10 and 15 mmHg. When using positive-pressure ventilation, a good strategy is to set the ventilator parameters to achieve a minimum mean airway pressure, moderate alkalosis (pH = 7.45, pCO_2_ = 35 mmHg), and maximum PVR [7], as was done in our case.

**Figure 3:**
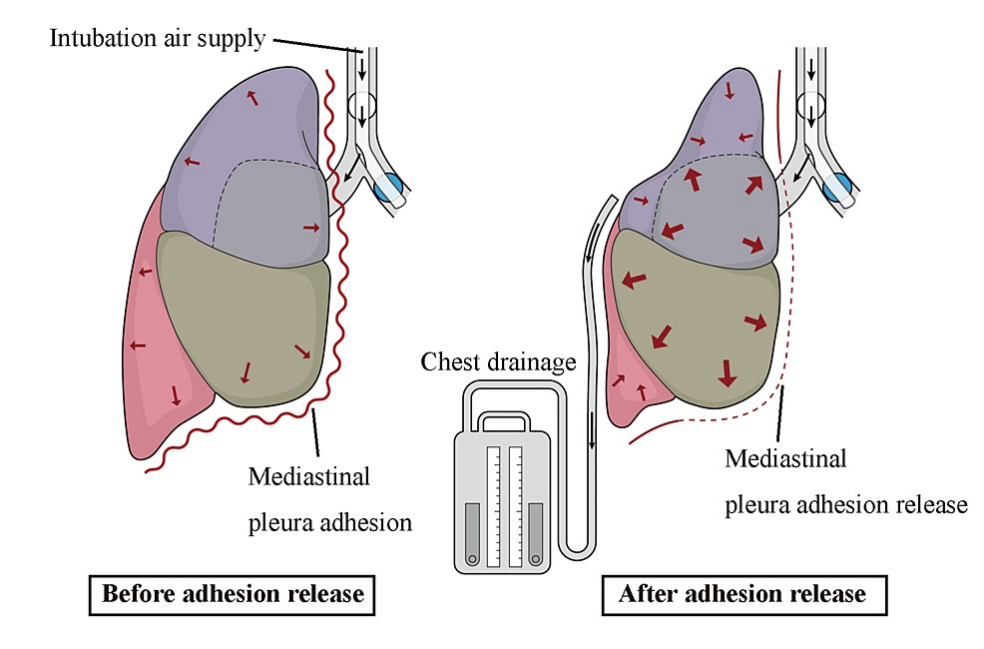
Hypothesis of the pathophysiology of this disease. Discussion of the pathophysiology of the condition in this case. Image credits: Mao Kinoshita.

Furthermore, during anesthesia management and thoracoscopic right lung suture in the post-Fontan procedure patient, the lungs did not dilate despite the absence of tracheal impassability. There have been no previous reports of such cases in patients with Fontan circulation. A thoracic drainage catheter was placed, and negative pressure was applied externally to dilate both lungs. In this case, with the patient’s pre-existing condition, excessively high airway pressure may have elevated intrathoracic pressure and interfered with ventricular venous return. Furthermore, it appeared difficult to approach the patient from within the body without obstructing tracheal pathways. The extent to which intra-airway pressure can be tolerated varies by patient, and no clinical trials have been reported. To maintain Fontan circulation, PVR should be kept low. Moreover, the patient may be vulnerable to respiratory complications, such as restrictive ventilation disorders, pulmonary arteriovenous fistula, diaphragmatic nerve palsy, and plastic bronchitis [8]. Therefore, following a consultation with a respiratory surgeon, a thoracic catheter was inserted, and negative pressure was applied from outside the body in a sealed position to ensure lower lobe expansion. The lack of lung inflation during the leakage test was attributed to increased lung compliance of the upper and middle lobes by releasing their adhesions to the anterior mediastinum, which reduced airflow to the dorsal parts of the lower and upper lobes due to the difference in respiratory resistance (Figure 3). Excessive airway pressure could not be applied to prevent an increase in PVR due to the Fontan circulation. This could be the cause of the failure to inflate lungs normally. The heterogeneity of lung inflation immediately after surgery due to disparities in airway resistance is not directly related to the Fontan circulation. However, the patient’s history of multiple chest surgeries and strong lung adhesions were noted to be contributing factors.

## Conclusions

In our case, a patient who had previously undergone Fontan surgery was managed under general anesthesia during a thoracoscopic right lung suture. Intraoperatively, one lung ventilation was performed with a left-sided double-lumen tube, and the lung was attempted to be dilated to check for air leaks but could not be dilated. A thoracic drain was inserted and negative pressure was applied externally to dilate both lungs, with successful and uncomplicated results. Patients after Fontan surgery require sensitive anesthetic management, including circulation and respiration, and we strongly recommend this approach in future practice and in similar patient populations.
